# Identification and Validation of SNP Markers Linked to Dwarf Traits Using SLAF-Seq Technology in *Lagerstroemia*

**DOI:** 10.1371/journal.pone.0158970

**Published:** 2016-07-12

**Authors:** Yuanjun Ye, Ming Cai, Yiqian Ju, Yao Jiao, Lu Feng, Huitang Pan, Tangren Cheng, Qixiang Zhang

**Affiliations:** Beijing Key Laboratory of Ornamental Plants Germplasm Innovation & Molecular Breeding, National Engineering Research Center for Floriculture, Beijing Laboratory of Urban and Rural Ecological Environment and College of Landscape Architecture, Beijing Forestry University, Beijing, 100083, China; Louisiana State University Agricultural Center, UNITED STATES

## Abstract

The genetic control of plant architecture is a promising approach to breed desirable cultivars, particularly in ornamental flowers. In this study, the F_1_ population (142 seedlings) derived from *Lagerstroemia fauriei* (non-dwarf) × *L*. *indica* ‘Pocomoke’ (dwarf) was phenotyped for six traits (plant height (PH), internode length (IL), internode number, primary lateral branch height (PLBH), secondary lateral branch height and primary branch number), and the IL and PLBH traits were positively correlated with the PH trait and considered representative indexes of PH. Fifty non-dwarf and dwarf seedlings were pooled and subjected to a specific-locus amplified fragment sequencing (SLAF-seq) method, which screened 1221 polymorphic markers. A total of 3 markers segregating between bulks were validated in the F_1_ population, with the M16337 and M38412 markers highly correlated with the IL trait and the M25207 marker highly correlated with the PLBH trait. These markers provide a predictability of approximately 80% using a single marker (M25207) and a predictability of 90% using marker combinations (M16337 + M25207) in the F_1_ population, which revealed that the IL and the PLBH traits, especially the PLBH, were the decisive elements for PH in terms of molecular regulation. Further validation was performed in the BC_1_ population and a set of 28 *Lagerstroemia* stocks using allele-specific PCR (AS-PCR) technology, and the results showed the stability and reliability of the SNP markers and the co-determination of PH by multiple genes. Our findings provide an important theoretical and practical basis for the early prediction and indirect selection of PH using the IL and the PLBH, and the detected SNPs may be useful for marker-assisted selection (MAS) in crape myrtle.

## Introduction

Controlling plant architecture is often a desirable goal in crop, horticultural and ornamental plants [1]. Such control is closely related to the yield and quality through its ability to interfere with the crop colony structure, the field microclimate and the solar energy utilization efficiency [2–4], although the ornamental value of plants is restricted because the plant architecture controls the spatial arrangement of various tissues and organs [5,6]. Dwarfism, which is regarded as one of the most important ornamental traits, has become a trend in new cultivar breeding because of its small crown, lodge resistance, increased production and convenient management [7,8].

The genetic mechanisms underlying plant height have been a consistent research focus since the introduction of lodging-resistant semi-dwarf rice and wheat mutants, which led to the ‘Green Revolution’ in the 1960s [9,10]. To date, the genetic inheritance of plant height and molecular marker development have been improved to a great extent, and a large number of genes related to dwarf traits have been cloned successfully [11–13]. Similar to the breeding programs of the dwarf cucumber and melon, numerous studies have been launched to dissect the genetic basis of traits associated with plant height [14–16]. Dwarfing rootstocks in apple trees are essential to ensure a greater yield per unit area over the life of the orchard [17]. *Dw1* is a major component of dwarfing apple trees, and it has been mapped between two markers by a bulked segregation analysis (BSA) and a genome scanning approach [18]. Dwarf plants play a unique role in enriching garden applications, and investigations that dissect the inheritance of dwarfing habits and the molecular markers linked to this trait should be performed to breed new cultivars with this plant type [19–21]. However, few studies on the architecture of ornamental plants have been reported.

*Lagerstroemia* (Lythraceae family) is native to southeastern Asia and Australia, where at least 50 species of these deciduous shrubs or small trees are found [22]. This genus was first cultivated in China approximately 1800 years ago [23], and certain species are widely used in gardens and regarded as an indispensable source of income for companies and retail nursery growers due to its graceful plant architecture, long-lasting summer bloom and rich colors [24]. Traditionally, *Lagerstroemia* species have been used as small trees or shrubs, although they have been recently bred for dwarf or potted plants. Dwarf crape myrtle cultivars are characterized by a low plant height, a compact plant type, short internodes and abundant mini flowers, and they have been selected for *Lagerstroemia* breeding programs [25]. To date, significant progress has been achieved with regard to new varietal breeding [26–28], germplasm evaluations [29], genetic diversity analyses [30,31], molecular marker development [32,33], genetic linkage map construction [34] and transcriptome analyses [35,36]. However, an in-depth study has not been conducted on the genetic mechanism underlying dwarf traits. Ye et al. [37] screened an AFLP marker using the F_1_ population of *L*. *fauriei* (non-dwarf) × *L*. *indica* ‘Pocomoke’ (dwarf), which was 23.33 cM from the loci controlling the dwarf traits. Investigations indicated that all of the polymorphic loci assayed within 15 cM of the target locus have been identified, and they gradually lose their effectiveness as the genetic distance increases [38]. Therefore, with a genetic distance of 23.33 cM from the dwarf genes, this AFLP marker may be ineffective at identifying phenotypes in other populations or cultivars. Regarding the weakness of current technologies in molecular marker development, it is difficult to obtain large amounts of markers to meet the requirement for breeding dwarf crape myrtle cultivars by marker-assisted selection (MAS).

In recent years, next-generation sequencing (NGS) technology has provided an effective method of developing numerous DNA markers in a short period. Initially, whole genome sequencing was just employed to identify genes in limited materials with a relatively small genome size [39]. However, this method is not effective for most materials that have a large genome and lack a reference genome sequence. Subsequently, Miller et al. [40] developed restriction site-associated DNA (RAD) markers, using for screening SNPs and genetic mapping in many plants such as barley and grape [41,42]. Peterson et al. [43] introduced a low-cost RAD sequencing (RADseq) technology referred to as double digest RADseq, which requires no prior genomic knowledge. After that, Poland et al. [44] reported a novel method called two-enzyme genotyping-by-sequencing (GBS), which is used to construct high-density genetic maps in many plants. This library construction technology greatly simplifies the quantification of the libraries prior to sequencing. The SLAF-seq (Specific Length Amplified Fragment Sequencing) technique is a high throughput, high accuracy, low cost method that has a short cycle, and it represents an efficient method of large-scale genotyping and was first described in Sun et al. [45]. In the procedure, massive specific-length SLAFs are obtained after a SLAF pre-design experiment. Then, pair-end sequencing is performed on the selected SLAFs using an Illumina high-throughput sequencing platform. Finally, the SLAF-seq data are analyzed by BLAT [46] to select specific fragments for the development of molecular markers. SLAF-seq technology has been tested on *Thinopyrum elongatum* and maize, and the data were strongly consistent between the predicted and virtual SLAFs [47,48]. With its high sequencing accuracy, this technology has broad applicability for molecular breeding, system evaluations and germplasm resource identification, and it should facilitate gene-mapping studies [49–52].

The present study aimed to dissert the inheritance of morphological traits and identify the SNP loci linked to dwarf genes in crape myrtle. To this end, the DNA from non-dwarf and dwarf individuals in a *L*. *fauriei* × *L*. *indica* ‘Pocomoke’ F_1_ segregating population was subjected to SLAF-seq. The linked markers were further validated both in the BC_1_ population and in a set of 28 crape myrtle materials by an allele-specific PCR (AS-PCR) analysis. The results of this study will contribute to further understanding the genetic determination of dwarf traits in the *Lagerstroemia* species.

## Materials and Methods

### Plant Materials

**Fig 1:**
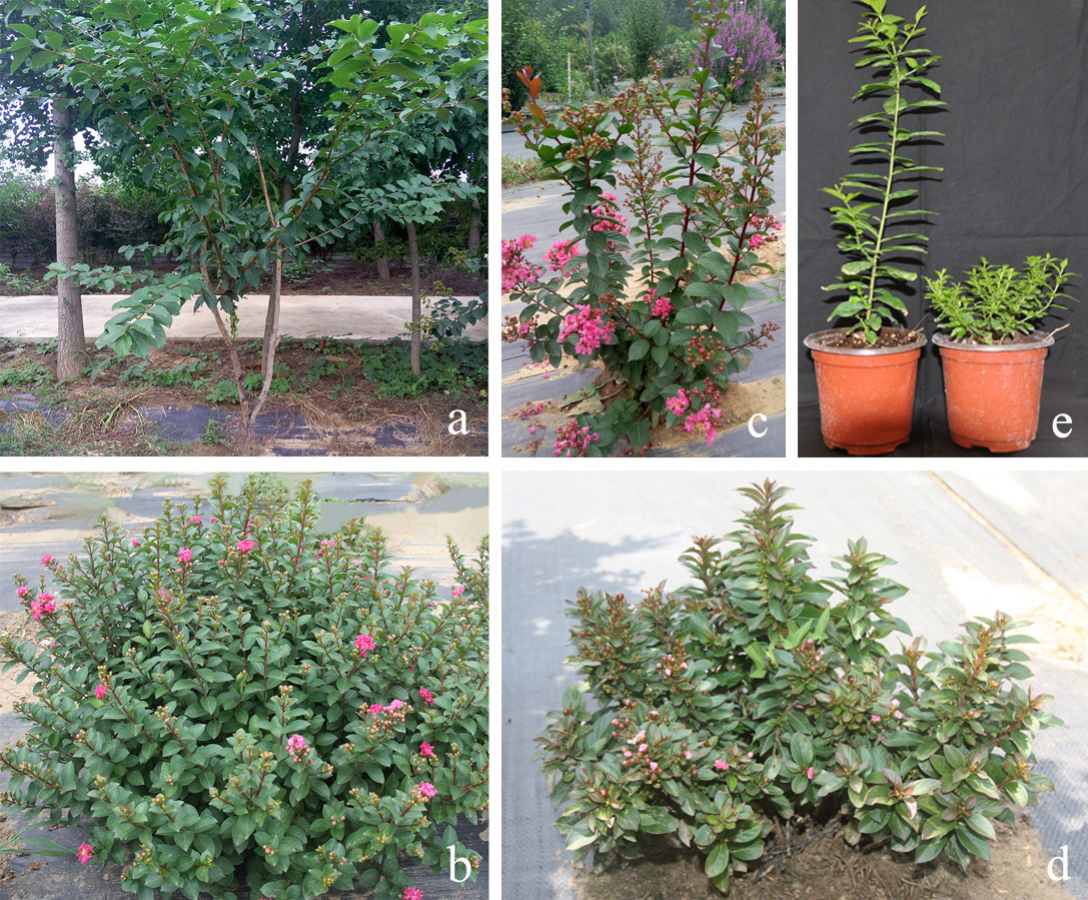
Plant materials used in this study. (a) Female parent, (b) male parent, (c) non-dwarf phenotype in the F_1_ population, (d) dwarf phenotype in the F_1_ population, and (e) two contrasting phenotypes in the BC_1_ population.

To identify the SNP markers linked to the dwarf phenotype in crape myrtle, the F_1_ segregating population was derived from a cross of *L*. *fauriei* (♀) × *L*. *indica* ‘Pocomoke’ (♂) in 2011. The crossing parents were selected for their contrasting plant architecture traits. The female parent was an arbor (> 3 m) with wide leaves and long internodes (Fig 1A), and the male parent was a dwarf shrub (0.3–0.6 m) with small leaves and short internodes (Fig 1B). Seedlings of the non-dwarf plant type in the F_1_ population (Fig 1C and 1D) were randomly selected for backcrossing with the male parent to generate the BC_1_ population during 2013 (Fig 1E). In addition to the F_1_ and BC_1_ populations, a set of 28 crape myrtle stocks were employed to further validate the association between the phenotypic traits and the SNP markers. In particular, the plant types of 28 crape myrtle stocks were defined to tree phenotype (height greater than 20 feet after 10 years), intermediate phenotype (height less than 20 feet after 10 years), semi-dwarf phenotype (height less than 12 feet after 10 years) and dwarf phenotype (height less than 4 feet after 5 years) [53]. The information on the species and cultivar along with their plant type and genetic background are described in He et al. [31]. Referring to the mean height of the non-dwarf seedlings, the dwarf-type seedlings were distinguished at half the height of the non-dwarf seedlings [37]. All of the materials were planted in an ornamental plant germplasm and a breeding nursery of the China National Engineering Research Center for Floriculture (CNERCF) (Beijing) (40°02′N, 115°50′E).

### Collection of phenotypic data

**Fig 2:**
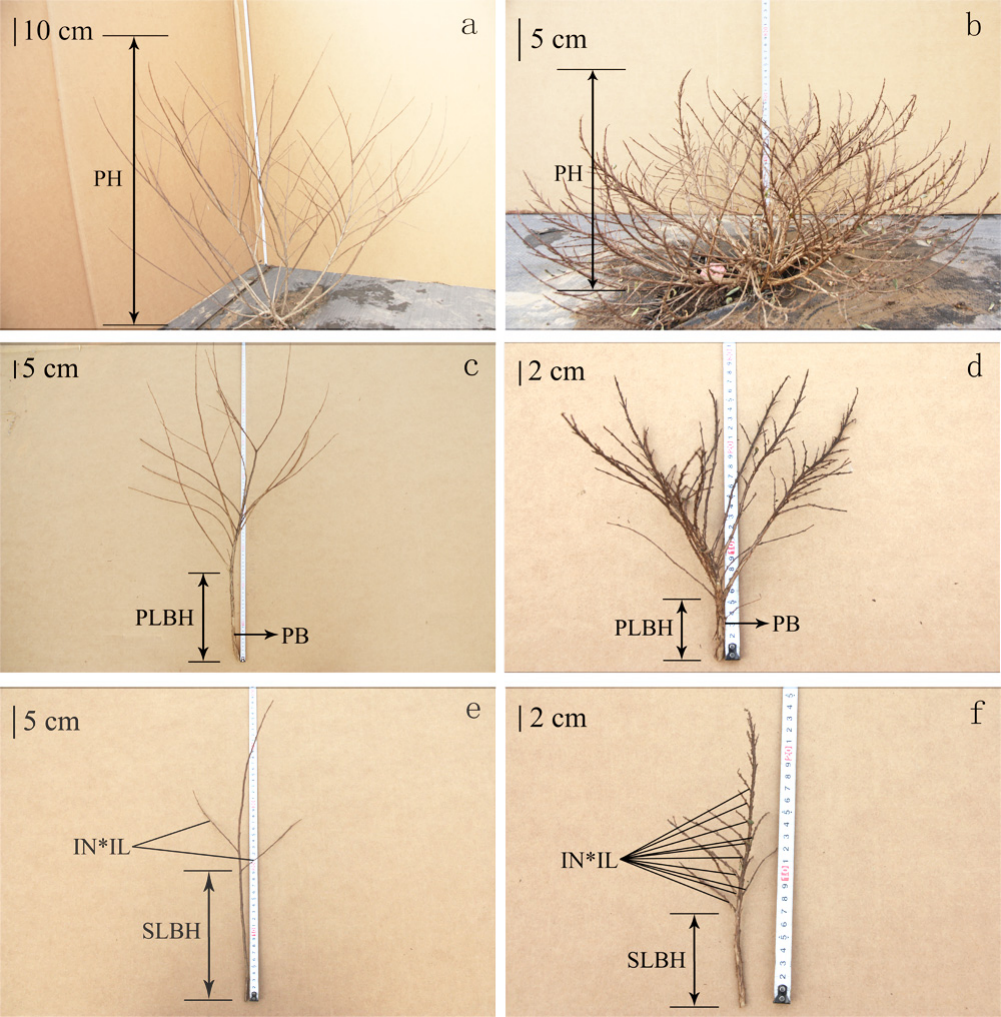
Pictorial representation of the phenotypic traits of dwarf and non-dwarf crape myrtle. PH, IL, IN, PLBH, SLBH and PBN represent plant height, internode length, internode number, primary lateral branch height, secondary lateral branch height and primary branch number, respectively. (a) PH for the non-dwarf phenotype, (b) PH for the dwarf phenotype, (c) PLBH and PB for the non-dwarf phenotype, (d) PLBH and PB for the dwarf phenotype, (e) IN, IL and PLBH for the non-dwarf phenotype, (f) IN, IL and PLBH for the dwarf phenotype.

A phenotypic trait assessment of 142 F_1_ hybrids was conducted at CNERCF during 2013 and 2014 under field conditions. At the end of the vegetative stage, six traits, including the plant height (PH), internode length (IL), internode number (IN), primary lateral branch height (PLBH), secondary lateral branch height (SLBH) and primary branch number (PBN), were investigated during the two consecutive years (Fig 2). The trails for the PH and PBN were evaluated for each individual with three technical replicates. We investigated other traits for each individual from three different orientations and selected annual branches to evaluate the IL and IN. An analysis of variance (ANOVA) was performed for the phenotypic data, and a Pearson correlation analysis and a linear regression were conducted using the SPSS Statistics 20.0 program (SPSS, Chicago, IL, USA).

### DNA extraction and pool construction

Genomic DNA was extracted from fresh young leaves using the FastDNA kit (Tiangen Biotech, Beijing, China) following the manufacturer’s protocol. The DNA was diluted to 50 ng/ul with an OD_260/280_ of 1.7–2.0. UV spectroscopy (NanoDrop ND-1000, Thermo Scientific, USA) was applied to examine the purity of the DNA samples and confirm the concentrations. Fifty plants each from non-dwarf and dwarf seedlings were randomly selected to construct the gene pools.

### SLAF fragment development by high-throughput sequencing

First, a pre-design SLAF experiment was performed according to the genome size, the GC content and the repeat sequence information of crape myrtle. The enzymes and sizes of the restriction fragments were evaluated using training data. Next, the SLAF libraries, including the parents and two gene pools, were constructed using the pre-design scheme. The SLAF sequencing procedure was performed as described by Sun et al. [45] with small modifications. Genomic DNA was digested into 450–500 bp fragments using suitable restriction enzyme combinations, including *EcoR*I + *Nla*III + *Mse*I. The restriction-ligation reactions were heat inactivated at 65°C and then digested with the additional restriction enzyme *Nla*III at 37°C. These reactions were diluted in 30 μl of elution buffer and mixed with dNTPs, Taq DNA polymerase (NEB, Ipswich, MA, USA), and *Mse*I-primer containing barcode 1 for a polymerase chain reaction (PCR). The PCR products were purified using E.Z.N.A. Cycle Pure Kit (Omega, UK) and incubated at 37°C with *Mse*I, T4 DNA ligase, ATP, and the Solexa adapter. Subsequently, the reaction products were purified using a Quick Spin column (Qiagen, Venlo, the Netherlands). The appropriate fragments with indexes and adaptors were isolated using a gel extraction kit (Qiagen). These fragment products were then subjected to PCR amplification with the Phusion Master Mix (NEB) and the Solexa Amplification primer mix to add barcode 2. The samples were gel purified, and 450–500 bp of DNA was excised and then diluted for Illumina sequencing. Precise monitoring was performed for each sequencing cycle, and the ratio of the high-quality reads with quality scores greater than Q20 (quality score of 20, indicating a 1% chance of an error and 99% confidence) in the raw reads and the GC content were calculated for quality control. During the entire process, the average sequencing depths were more than 20 fold in the parents and 100 fold in the progeny pools averagely. Sequence similarity was detected by BLAT [46], and sequences with over 90% identity were defined as a SLAF locus. In each of the SLAF loci, we examined the polymorphism locus between the parents. Then, all of the polymorphic SLAFs were genotyped in the progeny as well as in any offspring containing more than 80% of the SLAFs in the parents, i.e., 80% integrity of the SLAF markers in the individuals. Potential SLAFs with one genotype originating from M and the other from P were identified as markers.

### Data analysis of the SLAF-seq

We examined the validity of all of the markers by introducing the SNP-index [54]. The method to calculate the SNP-index is as follows: SNP-index(aa) = Maa/(Paa+Maa); SNP-index(ab) = Mab/(Pab+Mab); and Delta(SNP-index) = SNP-index(ab)-SNP-index(aa). In the above formulas, Maa indicates the depth of the aa population derived from *L*. *fauriei* (M), Paa indicates the depth of the aa population derived from *L*. *indica* ‘Pocomoke’ (P), Mab represents the depth of the ab population derived from M, and Pab represents the depth of the ab population derived from P. Markers located farther away from the dwarf genes indicate more significant the Delta (SNP-index) departures from 0, i.e., the closer the Delta (SNP-index) was to 1.0. Therefore, we employed markers with a Delta (SNP-index) > 0.3 as the potential markers.

### Verifying the markers using Sanger sequencing

From the potential SLAF markers, 30 non-dwarf and dwarf F_1_ seedlings were selected for validation. Based on each 80 bp read length of these sequences, PCR primers were designed for the amplification. The products were purified using a Quick Spin column (Qiagen) and then sequenced using Sanger technology. The SNPs were verified according the sequencing results between the parents and the individuals. The amplification reactions were performed at a volume of 25 μL, which contained 50 ng template DNA, 12.5 μL 2X *Taq* PCR Master Mix (Biomega, San Diego, CA, USA), 0.6 μL of each forward and reverse primer and 10.3 μL ddH_2_O. The PCR procedures were as follows: 94°C for 5 min; followed by 30 cycles of 94°C for 35 s; the appropriate annealing temperature (45°-60°C) for 30 s; 72°C for 1 min; and a final extension step at 72°C for 10 min.

### Association analysis by allele-specific PCR

The SNP primers for the allele-specific amplifications were designed as described by Bundock et al. [55] with minor modifications. Herein, we introduced mismatch base pairs from the 3’ end to increase the specificity of the primers. Two complementary primers were designed to anneal to the SNP at the 3’ end, with each primer annealing to a different allele (e.g., T-C and A-G). For each of the three markers (M16337, M25207, M38412), all of the samples in the F_1_ population were genotyped in duplicate 25 μL reactions that each contained approximately 50 ng template DNA using the following amplification procedures: 94°C for 5 min; followed by 30 cycles of 94°C for 35 s; the appropriate annealing temperature (45°-60°C) for 30 s; 72°C for 1 min; and a final extension step at 72°C for 10 min. Each individual genotype was observed by the banding pattern of the agarose gel photo, and then an association analysis between the SNPs and the six phenotype traits was performed based on the analysis of variance for the phenotypic traits. The predictability of the plant height using a single marker was evaluated by the consistency of the genotype-phenotype relationship [56], i.e., the association of a heterozygous SNP with the non-dwarf phenotype and a homozygous SNP with the dwarf phenotype. Finally, the marker combinations were also analyzed to improve the accuracy of the marker-assisted selection breeding program [57].

### Testing SNP markers in the BC_1_ population and commercial cultivars

Three SNP markers that are closely linked to dwarf traits were screened in the BC_1_ population (92 individuals) and a set of 28 commercial materials using AS-PCR technology. Herein, remarkable character separation of plant height was observed in the BC_1_ progenies, and the 28 commercial varieties were selected according to their diverse plant architectures.

## Results

### Statistical analysis of the phenotypic evaluation

**Fig 3:**
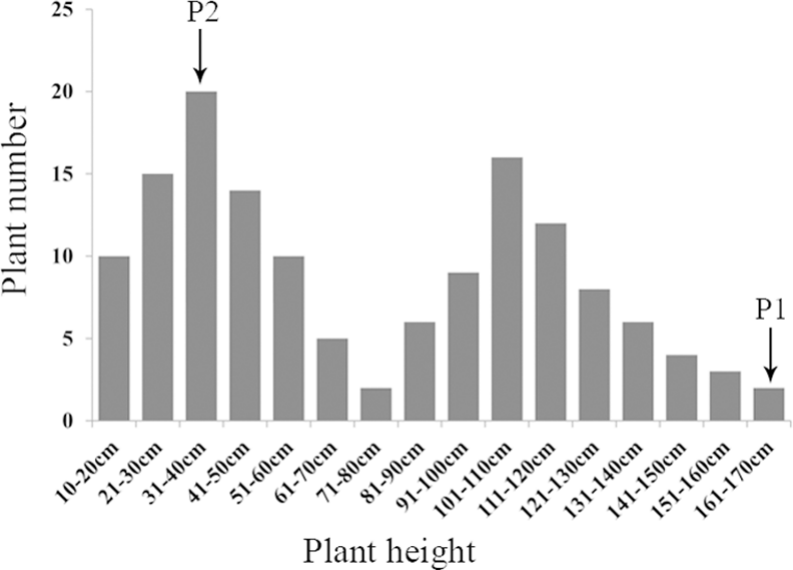
The frequency distribution of plant height in the F_1_ population and the parent lines. P1 represents the female parent and P2 represents the male parent.

**Fig 4:**
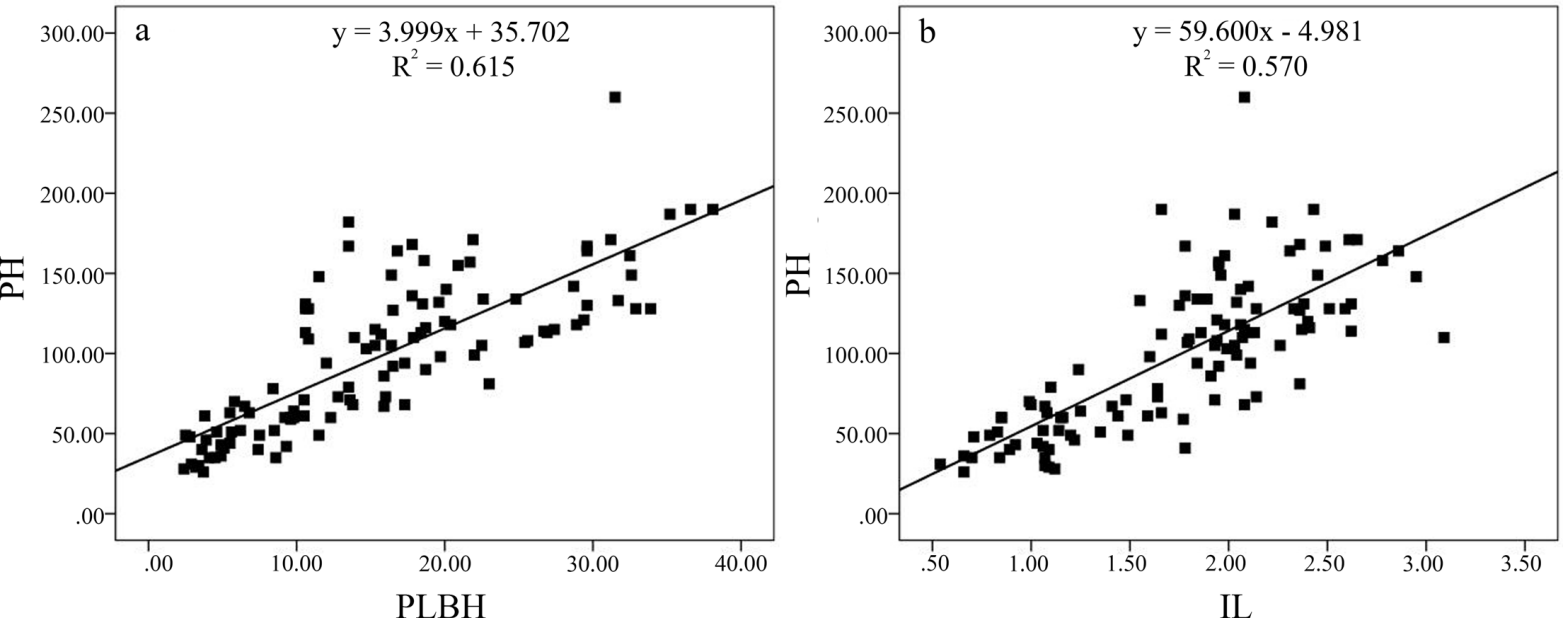
Scatter plots of the phenotypes in the F_1_ population derived from *Lagerstroemia fauriei* (non-dwarf) × *L*. *indica* ‘Pocomoke’ (dwarf). The traits were evaluated for each individual with three replicates. All of the data analyses were conducted using the SPSS Statistics 20.0 program (SPSS, Chicago, IL, USA); P < 0.01. (a) PH and IL, and (b) PH and PLBH.

We observed six phenotypic traits during 2013 and 2014 in the F_1_ population. The descriptive statistics of the plant architecture traits for the parents and the F_1_ population during two consecutive years are presented in Table 1. Overall, the F_1_ progenies exhibited a higher coefficient of variation for PH, PLBH and IL than IN, SLBH and PBN in the two years. The frequency distribution of the plant height in the F_1_ and parent lines showed that the PH was controlled by a major gene plus polygenes and was suitable for the bulked segregant analysis (Fig 3), which was consistent with previous work [37]. Each phenotypic trait between the 2 years was significantly correlated with a correlation coefficient > 0.9 (P < 0.01). The Pearson correlation coefficients between the six traits are described in Table 2. The PH trait showed a significant positive correlation with the IL, PLBH, and SLBH and a negative correlation with the PBN. Although we evaluated the IL, IN, PLBH, SLBH and PBN as different traits to determine the PH, only the IL and the PLBH could be used as representative indexes of PH. The scatter plots are shown in Fig 4. The R^2^ values between the PH and IL and between the PH and PLBH were 0.570 and 0.615, respectively, which indicated that early predictions and indirect selection for plant height using IL and PLBH are practical for use in crape myrtle breeding programs.

**Table 1:** Descriptive statistics of six traits for *L*. *fauriei* (P1) × *L*. *indica* ‘Pocomoke’ (P2) and the F_1_ population over 2 years (2013 and 2014).

Trait	Parent		F_1_ population				
	P1	P2	Mean	SD	Min	Max	CV
2013 PH (cm)	160.5	31.3	66.3	41.2	14.2	159.9	62.1%
2014 PH (cm)	168.9	32.6	72.8	45.7	18.8	170.1	62.8%
2013 IL (mm)	2.7	0.7	13.9	6.8	0.5	2.8	48.9%
2014 IL (mm)	2.6	0.7	13.5	6.6	0.6	3.0	48.9%
2013 IN	8.0	16.0	14.1	1.3	7.0	19.0	9.2%
2014 IN	8.0	17.0	13.6	1.4	7.0	21.0	10.3%
2013 PLBH (cm)	16.8	5.1	14.3	8.5	4.9	21.1	59.4%
2014 PLBH (cm)	18.7	5.6	15.0	8.9	4.2	22.7	59.3%
2013 SLBH (cm)	13.4	6.9	14.9	3.5	6.4	20.0	23.4%
2014 SLBH (cm)	15.1	7.2	15.6	3.2	6.1	20.8	20.5%
2013 PBN	3.0	9.0	9.8	0.8	4.0	14.0	8.2%
2014 PBN	4.0	8.0	12.1	1.0	4.0	16.0	8.3%

**Table 2:** Pearson correlation coefficients between the plant architectural traits in the F_1_ population.

Trait	PH	IL	IN	PLBH	SLBH	PBN
PH	1					
IL	0.784**	1				
IN	-0.162	-0.539**	1			
PLBH	0.819**	0.641**	-0.157**	1		
SLBH	0.466**	0.505**	-0.158**	0.363**	1	
PBN	-0.573**	-0.630**	0.234**	-0.574**	-0.389**	1

** indicates a significant correlation at *P* < 0.01.

### Analysis of the SLAF-seq data

A total of 3.83 Gb raw data was acquired using the SLAF-seq technology, and it contained 32,154,654 valid single-end reads with a read length of 80 bp (Table 3). The GC (guanine-cytosine) content was 39.99%, and the Q20 ratio (a quality score of 20) was 88.84%. The number of SLAF tags was 79,863, and the average coverage for each tag was 197.9 fold. The average sequence depth of the SLAF fragments was at least 20 fold in the parents and 50 fold in each of the progeny pools. Lacking for the genomic information of crape myrtle, we used the same restriction enzyme combination on the genome of *Eucalyptus grandis* and drew distribution diagram of SLAF tags on 11 scaffolds more than 35M [58]. The crape myrtle genome was successfully simplified because the SLAFs distributed equally on each chromosome (S1 Fig). According to the population information and previous research results, the dwarf trait of crape myrtle exhibits a quantitative character that is controlled by a major gene and modified by minor genes [37]. We hypothesized that the traits were controlled by *Ff/ff*; thus, the genotypes of the P, M, dwarf pool and non-dwarf pool at the polymorphic site were *ff*, *Ff*, *ff* and *Ff*, respectively, which simplified the model. Based on the SNP-index analysis, 38 SLAF tags were identified as sequences related to the dwarf traits.

**Table 3:** Sequencing data of each sample.

Sample	BMK-ID	Read Length (bp)	Read Number	GC Percentage
Male parent	P	80+80	4034530	40.28%
Female parent	M	80+80	3969057	40.43%
Dwarf pool (n = 50)	ab	80+80	12864866	40.22%
Non-dwarf pool (n = 50)	aa	80+80	11286201	39.87%

### Marker development for dwarf traits

**Fig 5:**
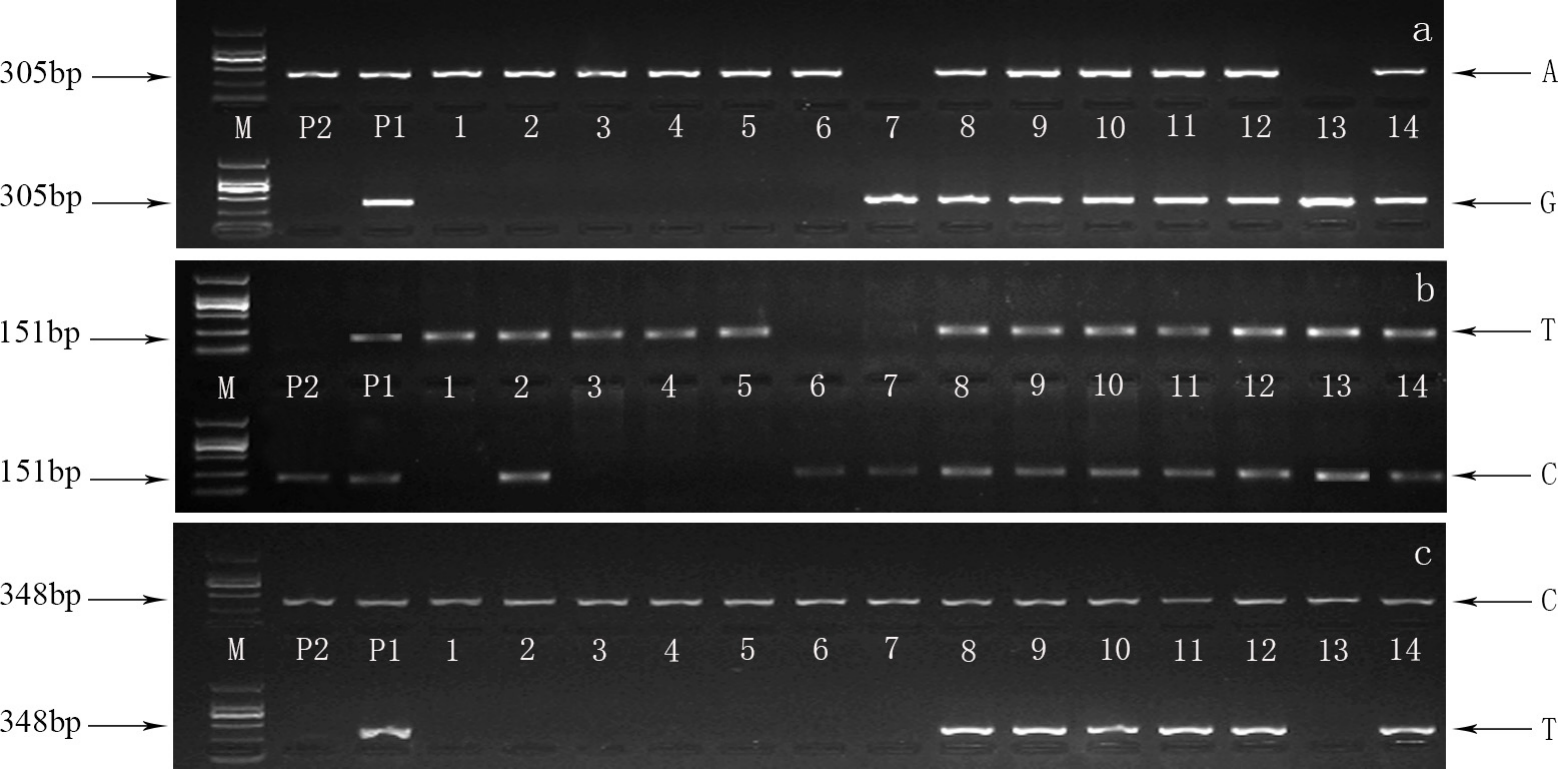
Agarose gel photo of the allele specific (AS) amplifications of the three markers. a, b and c PCR products of M16337, M25207 and M38412, respectively. M represents a 2000 bp marker followed by the parent lines and lanes 1–14 with template DNA derived from: P1 female parent, P2 male parent, 1–7 dwarf type individuals, 8–14 non-dwarf type individuals. The same samples were employed in the same order between the upper and lower band of the AS-PCRs. For the three markers, the upper band and lower band were amplified independently of the targeted SNP genotype. The corresponding tested SNP alleles are indicated in the figure.

Thirty-eight pairs of primers were designed to develop the specific molecular markers based on the related sequences. The PCR products were amplified from 30 extremely non-dwarf and dwarf F_1_ seedlings. A total of three specific markers was acquired using Sanger sequencing (Table 4) and the SNP-index of the three markers were described in Table 5. The results showed that the virtual SNPs were the same as those indicated in the SLAF-seq. Completing the full length of the SLAF sequence (S1 Table) allowed us to design primers to perform the association analysis using an allele-specific genotyping assay. Consequently, three markers were successfully developed to interrogate the SNPs (Table 6), and they were specific, stable, and repeatable in the F_1_ population. We show here that the AS-PCR technology is a considerably reliable method for genotyping each individual (Fig 5). The agarose gel photo of the three markers shows that the male parent amplified only one band and the female parent amplified two bands, which indicates that the corresponding SNP was homozygous and heterozygous, respectively. In addition, seedlings from the two contrasting phenotypes exhibited considerable genotype consistency. In total, three genotypes were identified in M16337 (AA/GG/AG) (Fig 5A) and M25207 (CC/TT/CT) (Fig 5B) and two genotypes were identified in M38412 (CC/CT) (Fig 5C), which was consistent with the performance by Sanger sequencing.

**Table 4:** Primer sequences of the specific molecular markers used in the Sanger sequencing.

Primer Name	Sequences of the Special Primers (5’-3’)		PCR Product	Original Fragment
	Forward	Reverse		
Dw_1	CCGTGATAATAATGGTAG	ATGTGAGTCATTGTGGAT	439 bp	M16337
Dw_2	GTAGACAGACGTATACAGC	CGTTGACATTGCCAC	448 bp	M25207
Dw_3	GATGCCACCTGAAGTTATT	GATTTTCCGGCGACTC	389 bp	M38412

**Table 5:** SNP-index information of the three markers.

Marker ID	Mab	Pab	Maa	Paa	SNP-index (aa)	SNP-index (ab)	Delta (SNP-index)
M16337	86	42	20	147	0.1198	0.6719	0.5521
M25207	7	29	39	0	1	0.1944	0.8056
M38412	20	29	57	0	1	0.4082	0.5918

**Table 6:** Primer sequences of the AS-PCR and the SNP loci.

Primer Name	Sequences of the Special Primers (5’-3’)			PCR Product	Original Fragment
	Forward	Reverse1	Reverse2		
AS_1	TCCCGTGGCTCTAACCTCT	TCTTGAACCATTTTTTTCCCT	TCTTGAACCATTTTTTTCCCC	305 bp	M16337
AS_2	TAGAACAAGACTCGGAAAA	CGCACATCGTACGTAAAA	CGCACATCGTACGTAAAG	151 bp	M25207
AS_3	CCAACGAGCAGCATCCAAAG	GGCGACTCGAACTTCTCCG	GGCGACTCGAACTTCTCCA	348 bp	M38412

### Genotype-phenotype association analysis

**Fig 6:**
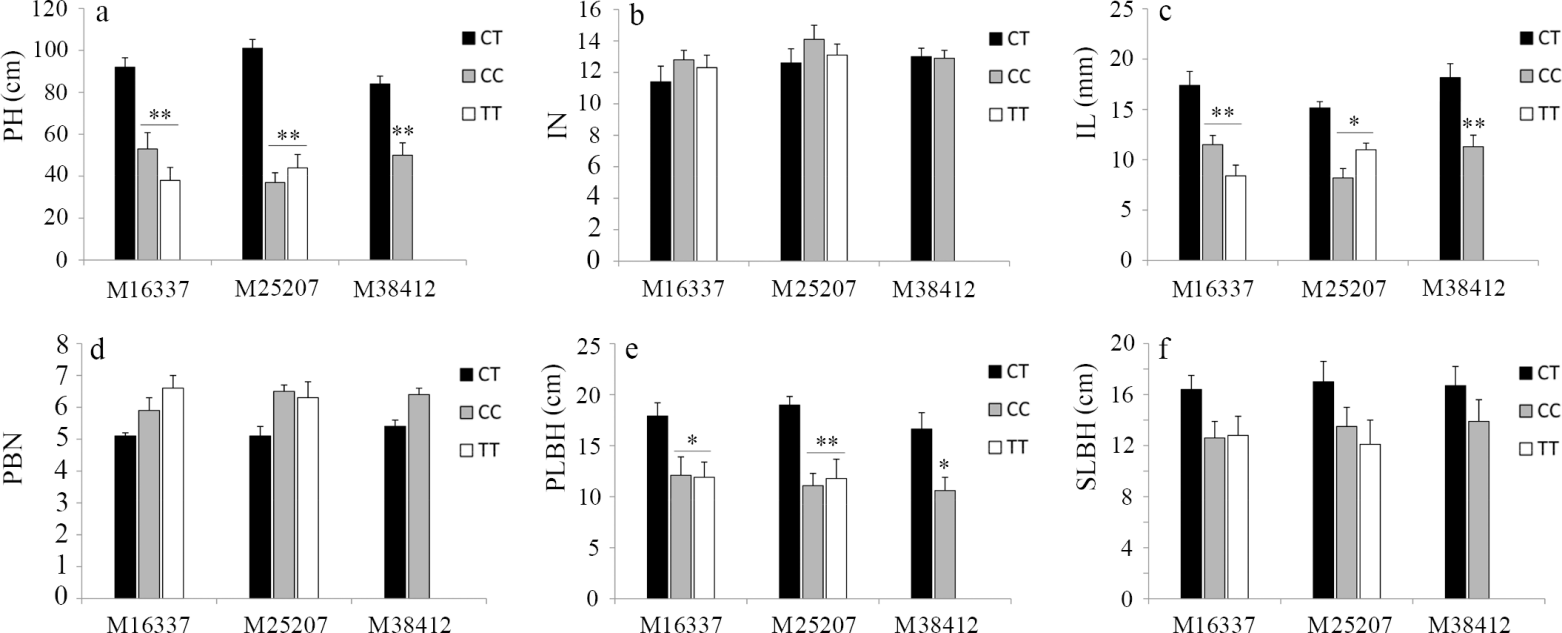
Analysis of the phenotype variance corresponding to the three markers in the F_1_ population (one-way ANOVA). All of the abbreviations are the same as above. The X-axis represents the three SLAF markers, and the Y-axis represents the phenotypic traits in the F_1_ population. (a) Correlation analysis between PH and the three markers, (b) correlation analysis between the IN and the three markers, (c) correlation analysis between the IL and the three markers, (d) correlation analysis between the PBN and the three markers, (e) correlation analysis between the PLBH and the three markers, (f) correlation analysis between the SLBH and the three markers. Pillar colors represent the different genotypes of each marker as follows: black pillar, CT; grey pillar, CC; and white pillar, TT. Asterisks indicate P < 0.05 (*) and P < 0.01 (**) according to Student’s *t*-test.

The Pearson correlation analysis indicated that M16337, M25207 and M38412 were significantly correlated with PH (P < 0.01) (Fig 6), M16337 and M38412 were highly correlated with the IL (P < 0.01), and M25207 was highly correlated with the PLBH (P < 0.01). However, the expected association was not observed between the three markers and the IN, PBN and SLBH. Combined with the statistical analysis of the phenotypic evaluation, we speculated that PH is mainly controlled by the value of the IL and the PLBH; thus, PH can be predicted by the three markers in breeding programs.

**Fig 7:**
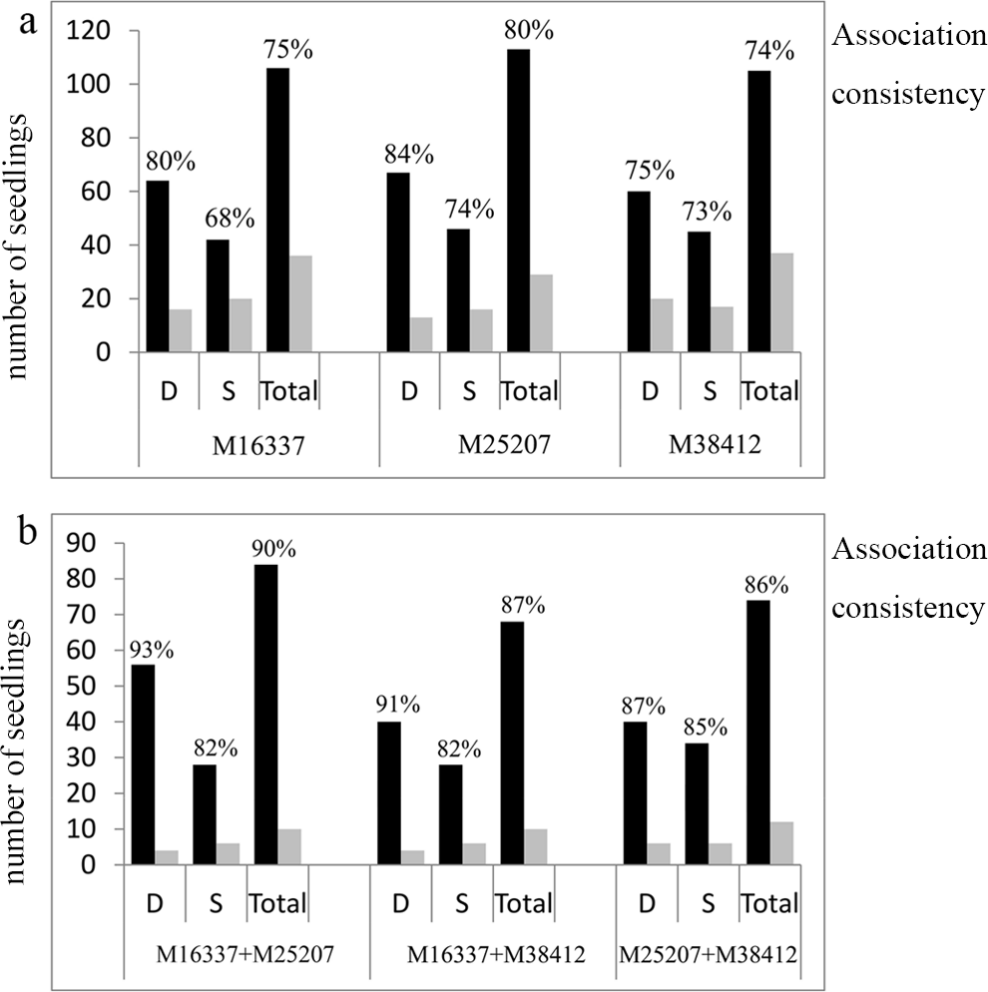
Association consistency between the plant architecture phenotypes and the SNP markers in the F_1_ population. The figures immediately above each column are the accuracy rates observed in that genotype-phenotype category. Association consistency is the proportion of seedlings for each plant type phenotype with the expected genotype. The black pillar represents the correct proportion, and the grey pillar represents the wrong proportion in the genotype-phenotype association. (a) Association consistency using a single marker, and (b) association consistency using marker combinations.

Consistency of the genotypes with the phenotypic traits for the three markers was observed in 142 F_1_ seedlings (Fig 7). Overall, at least 74% of the individuals presented the expected association between the SNPs and the dwarf traits, and the accuracy rate using M25207 was 80% (113/142 seedlings). Of all of the markers, a higher association rate was detected in the dwarf seedlings than in the non-dwarf seedlings, indicating increased ambiguity in the phenotype identification with the heterozygous genotype. The association rate between M25207 and the dwarf seedlings was 84% (67/80 seedlings), whereas the rate between M16337 and the non-dwarf seedlings was only 68% (42/62 seedlings). However, we found that the prediction accuracy was remarkably improved using different marker combinations. The most efficient combination was M25207 + M16337, which had a consistency rate of 89% (84/94 seedlings), wherein 93% of the seedlings were associated with the dwarf phenotypes and 82% of the seedlings were associated with the non-dwarf phenotypes, respectively. The combinations M25207 + M38412 and M16337 + M38412 provided approximately 86% and 87% predictability in the progeny.

### Testing SNP markers in the BC_1_ population and commercial cultivars

**Fig 8:**
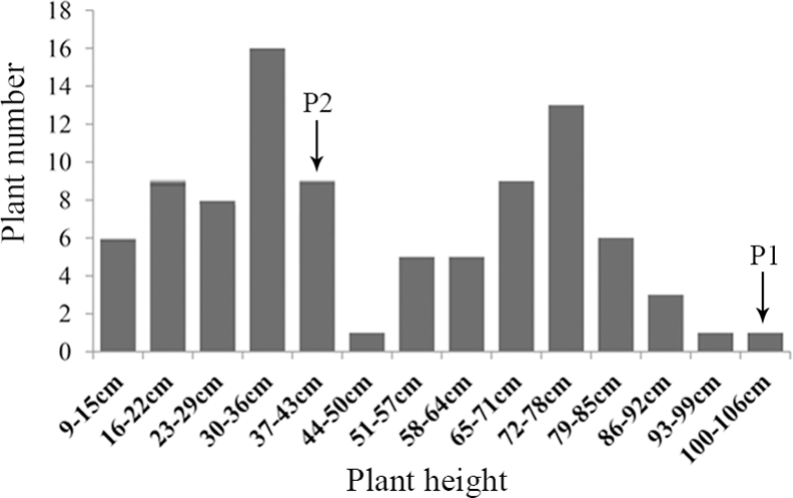
Frequency distribution of plant height in the BC_1_ population and the parent lines. P1 represents the female parent (non-dwarf seedling in the F_1_ population) and P2 represents the male parent (*L*. *indica* ‘Pocomoke’).

**Fig 9:**
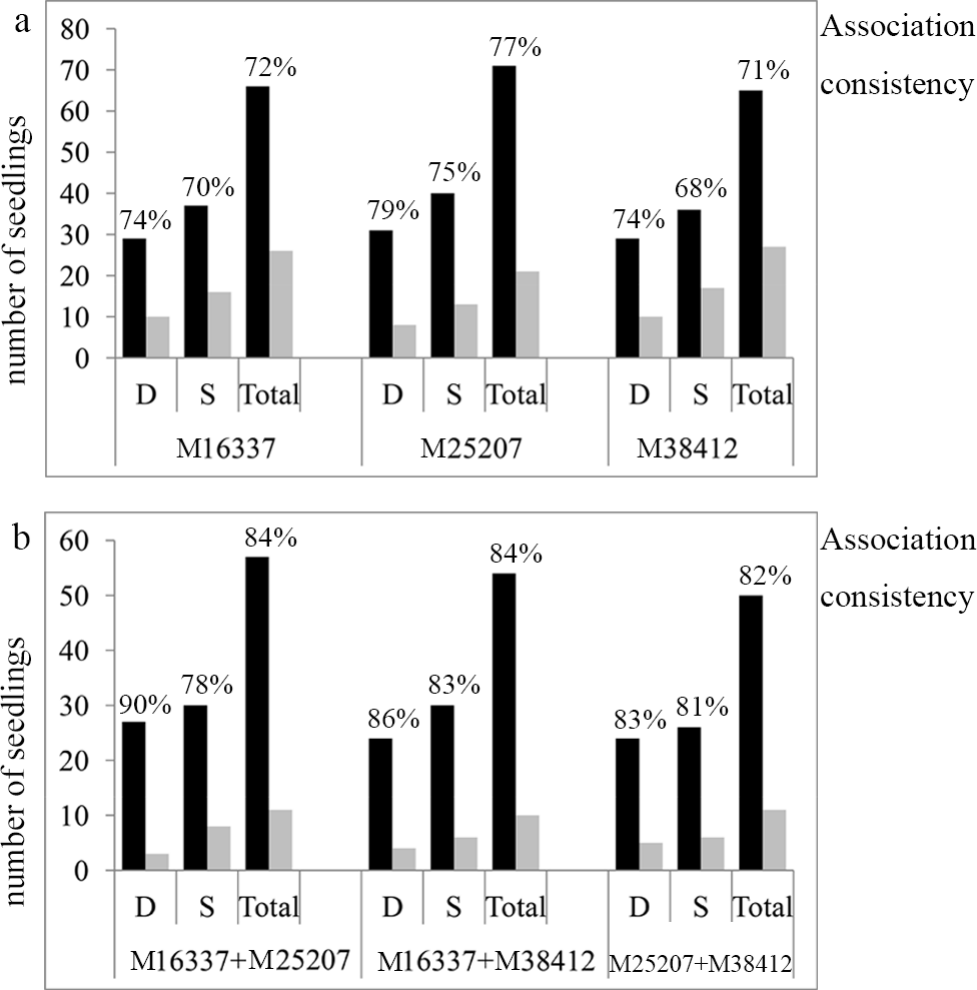
Association consistency between the plant architecture phenotypes and the SNP markers in the BC_1_ population. The figures immediately above each column are the accuracy rates observed in that genotype-phenotype category. The association consistency is the proportion of seedlings for each plant type phenotype with the expected genotype. The black pillar represents the correct proportion, and the grey pillar represents the wrong proportion in the genotype-phenotype association. (a) Association consistency using a single marker, and (b) association consistency using marker combinations.

The frequency histogram of the plant height in the BC_1_ population was shown in Fig 8. To test the validity of the three SNP loci for MAS breeding, a BC_1_ population with 92 seedlings was assessed by an AS-PCR analysis. The efficiency at which the three markers were able to identify the different phenotypes decreased slightly, although it still provided at least a 71% predictability using a single marker (M38412) and an 82% predictability using marker combinations (M25207 + M38412) (Fig 9). The results indicated a range of genotypes of the three markers in the various progenies with plant type separation, particularly in breeding programs in which the L. *fauriei* × *L*. *indica* ‘Pocomoke’ were the parents. The marker association patterns were further confirmed for a set of 28 commercial varieties representing diverse plant architectures (Table 7). In all of the tested samples, none of the markers precisely distinguished the phenotype. However, an interesting scenario was observed between the marker combinations and the plant type. When homozygotes or heterozygotes were screened simultaneously using three marker combinations, the tested sample exhibited the dwarf or tree phenotype, respectively. When one or two homozygotes were screened simultaneously, the tested sample exhibited the intermediate or semi-dwarf phenotype, respectively. Combining the three markers is an effective method to identify the plant phenotype, i.e., the genotypes of the three markers co-determined the plant height in the *Lagerstroemia* species.

**Table 7:** Testing the SNP markers in a set of 28 *Lagerstroemia* stocks with diverse plant architectures.

Species and cultivars	Plant type	Genetic background	M16337	M25207	M38412
*L*. *faurei*	tree	*L*. *faurei*	CT	CT	CT
*L*. *indica* ‘Catawba’	intermediate	*L*. *indica*	CC	CT	CT
*L*. *indica* ‘Comanche’	intermediate	*L*. *indica* 3/4, *L*. *faurei* 1/4	CT	CT	CC
*L*. *indica* ‘Yuma’	intermediate	*L*. *indica* 3/8, *L*. *faurei* 3/8, *L*. *amabilis* 1/4	CT	TT	CT
*L*. *indica* ‘Pecos’	semi-dwarf	*L*. *indica* 1/2, *L*. *faurei* 1/2	TT	CC	CT
*L*. *indica* ‘Acoma’	semi-dwarf	*L*. *indica* 3/4, *L*. *faurei* 1/4	TT	CC	CT
*L*. *indica* ‘Victor’	semi-dwarf	*L*. *indica*	CT	TT	CC
*L*. *indica* ‘Centennial’	semi-dwarf	*L*. *indica*	CT	CC	CC
*L*. *indica* ‘Prairie Lace’	semi-dwarf	*L*. *indica*	CC	CC	CC
*L*. *indica* ‘Tonto’	semi-dwarf	*L*. *indica* 3/4, *L*. *faurei* 1/4	CC	CC	CT
*L*. *indica* ‘Baton Rouge’	semi-dwarf	*L*. *indica*	CC	CT	CC
*L*. *indica* ‘Bayou Marie’	semi-dwarf	*L*. *indica*	CT	CC	CC
*L*. *indica* ‘Cordon Blue’	semi-dwarf	*L*. *indica*	CC	CC	CT
*L*. *indica* ‘Purple Velvet’	semi-dwarf	*L*. *indica*	CT	CC	CC
*L*. *indica* ‘Mardi Gras’	dwarf	*L*. *indica*	CC	CT	CC
*L*. *indica* ‘Okmulgee’	dwarf	*L*. *indica*	CT	CC	CC
*L*. *indica* ‘Velma’s Royal Delight’	dwarf	*L*. *indica*	TT	TT	CC
*L*. *indica* ‘New Orleans’	dwarf	*L*. *indica*	CC	CC	CC
*L*. *indica* ‘Sacramento’	dwarf	*L*. *indica*	CC	CC	CC
*L*. *indica* ‘Delta Blush’	dwarf	*L*. *indica*	TT	CC	CC
*L*. *indica* ‘Lafayette’	dwarf	*L*. *indica*	CC	CC	CC
*L*. *indica* ‘Houston’	dwarf	*L*. *indica*	CC	CC	CC
*L*. *indica* ‘Bourbon Street’	dwarf	*L*. *indica*	CT	CC	CC
*L*. *indica* ‘Creole’	dwarf	*L*. *indica*	CC	CC	CC
*L*. *indica* ‘Chisam Fire’	dwarf	*L*. *indica*	CC	CC	CC
*L*. *indica* ‘Chickasaw’	dwarf	*L*. *indica* 5/8, *L*. *faurei* 3/8	CC	CC	CC
*L*. *indica* ‘Pocomoke’	dwarf	*L*. *indica* 5/8, *L*. *faurei* 3/8	CC	CC	CC

## Discussion

Dwarfism in several crop plants is a commercially important production trait and formed the basis of the ‘Green Revolution’ in certain countries [9,10,59]. In addition, dwarfing traits, such as stem length in *Cucumis melo* [15], short internodes in *Cucumis sativus* [14] and bush-type growth habits in *Brassica napus* [60], have been reported in many horticultural plants. In the *Lagerstroemia* species, dwarf phenotype progenies show additional internode numbers, shorter internode lengths and intricate lateral branches. Identifying functional markers or key genes related to this complicated trait is a challenge. In addition, because of the lack of genomics information, it is difficult to obtain large amounts of markers to meet the requirement for breeding dwarf crape myrtle cultivars by MAS. Consequently, the inheritance of dwarf traits in crape myrtle has not been clearly characterized to date, which has directly resulted in the sluggish progress of dwarfing breeding and new cultivar development.

According to Ye et al. [37], 41 SSR and 384 AFLP primers were employed to map target genes using the bulked segregant analysis method. Nevertheless, the results showed that only the AFLP marker M53E39-92 was screened, and it was 23.33 cM from the loci controlling the dwarf trait. Regarding the relatively large genetic distance, additional works are required to identify the molecular markers that are tightly linked to the dwarf genes (< 10 cM) for practical breeding programs. SLAF-seq technology was developed according to a high-throughput sequencing technology, and it provides a new method of managing whole genome density distributions from large amounts of sequences [45]. This high-throughput, high-accuracy, low-cost and short-cycle technology can perform fine mapping of target genes within dense genetic information [61–63]. In this study, 1221 polymorphic SLAF markers were obtained using the SLAF-seq technology, and 38 specific markers were developed based on the SNP-index association analysis. Additionally, the data shows that the SLAF-seq technology is 1/8 the cost of AFLP technology and 27-times more efficient (http://www.biomarker.com.cn/). Therefore, compared with traditional markers, such as RAPD, AFLP, ISSR and SSR, the efficiency of SLAF-seq technology is much better for developing plant molecular markers.

The F_1_ population employed in the phenotype evaluation had an appropriate number of individuals; therefore, the Pearson correlation analysis and linear regression analysis were accurate. The genetic analysis showed that the IL and the PLBH were positively correlated with PH; therefore, these traits could be considered a representative index of PH, indicating that this compact trait should be co-determined by multiple genes instead of one single gene.

In the present study, three markers were successfully employed to assay for SNPs using the AS-PCR technology, which was highly stabile and repeatable. The genotypes were consistent with the dwarf trait in the F_1_ population, and the three markers exhibited precise accuracy in the genotype-phenotype association. We found that M25207 showed a higher overall association rate than the other two markers, indicating that the PLBH might be a more accurate indicator when altering plant types. Among all of the markers, a higher association rate between the SNPs and the phenotype was detected in the dwarf seedlings than in the non-dwarf seedlings. Compared with the F_1_ population, the efficiency at which the three markers were able to identify different phenotypes decreased slightly in the BC_1_ population. Progenies from the F_1_ population were selected for the gene pool construction using SLAF-seq technology and resulted in a higher association rate relative to the other populations. Although the genotypes were identified accurately, the association analysis was prone to errors if inaccurate phenotype identifications are included. The population of BC_1_ individuals includes 2-year-old seedlings; thus, the characteristics of the plant architecture have not been finalized, which may have caused discrepancies in the consistency between the markers and the phenotypes.

Combinations of the markers associated with the phenotypes were superior to that of single markers. The accuracy was remarkably improved using different marker combinations, and the M25207 + M16337 combination provided an approximately 90% predictability in the F_1_ progeny, thereby indicating an effective method of screening dwarf plants in marker-assisted selection breeding programs for crape myrtle. The results were also tested using a set of 28 *Lagerstroemia* stocks with diverse plant types, which revealed the co-determination of plant height by the three markers.

In recent years, molecular markers have been widely studied in crop breeding programs [64]. MAS improves the breeding efficiency and accelerates the breeding process by using DNA markers that are tightly linked to the target genes [56]. Therefore, to breed new crape myrtle varieties with exquisite plant architectures, it is important to develop molecular markers that are closely linked to the dwarf traits. Our results indicated that M25207 and M25207 + M16337 provided an 84% and 93% prediction rate, respectively, which may be an acceptable level of reliability in most breeding programs, particularly those aimed at selecting seedlings with a dwarf plant height or a short internode. However, linked markers rather than the genes themselves were identified in this study, and this process presents certain limitations in breeding practice. Therefore, additional work is required to perfect the genotypic prediction of dwarf traits.

Several scenarios may have occurred within those individuals who did not present the expected genotype-phenotype association. As discussed above, multiple potential phenotypic traits likely co-regulate the plant architecture type, such as the internode length, the internode number and the lateral branch [5,65,66]. Such plant architectures are controlled by genetic regulation, including genes and hormones [67,68], and they are also adjusted by environmental factors [69]. The plant height trait in this research exhibited continuous variance, indicating that this characteristic is quantitative. Nevertheless, SLAF-seq technology is a method based on bulked segregant analyses (BSAs), and it may not be ideal for identifying quantitative traits, such as plant height, crop yield and disease resistance. Future studies of crape myrtle should focus on QTL mapping for plant height, internode length, internode number and lateral branch to dissert tree architectural plasticity into genetic, ontogenetic and environmental effects.

## Supporting Information

S1 Fig The distribution diagram of SLAF tags on the genome of *Eucalyptus grandis*. (TIF)

S1 Table The DNA sequences and length of the specific markers related to dwarf traits in crape myrtle. (DOCX)
