# Supplementary figures and images for: Identification and Validation of SNP Markers Linked to Dwarf Traits Using SLAF-Seq Technology in Lagerstroemia
Source: PLoS One. 2016 Jul 12;11(7):e0158970. doi: 10.1371/journal.pone.0158970 (PMC4942086; doi:10.1371/journal.pone.0158970)

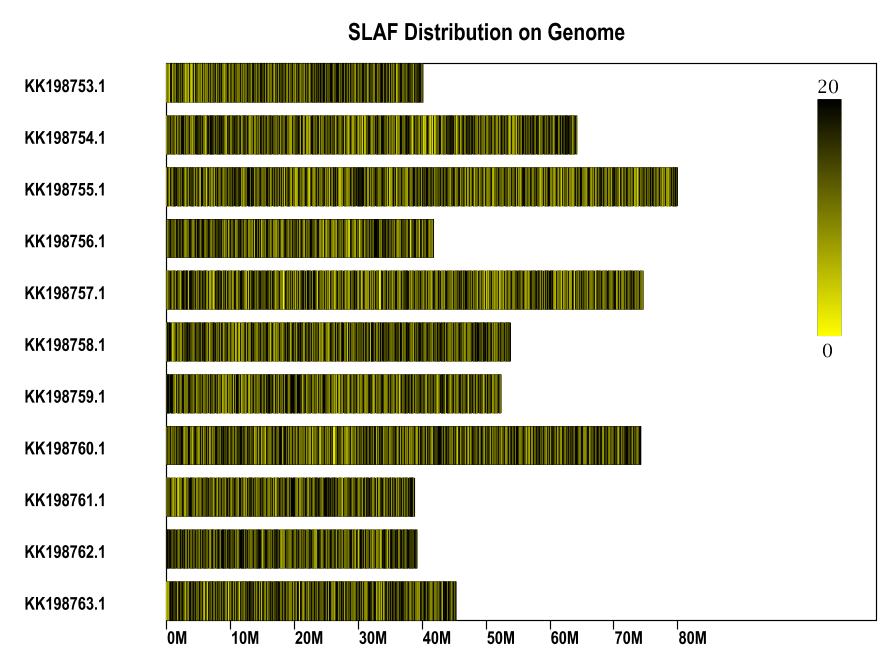

Supplement: S1 Fig — (TIF) [file pone.0158970.s001.tif]
