# Supplementary material for: Identification and Validation of SNP Markers Linked to Dwarf Traits Using SLAF-Seq Technology in Lagerstroemia
Source: PLoS One. 2016 Jul 12;11(7):e0158970. doi: 10.1371/journal.pone.0158970 (PMC4942086; doi:10.1371/journal.pone.0158970)
Supplement: S1 Table — (DOCX) [file pone.0158970.s002.docx]

**S1 Table. The DNA sequences and length of the specific markers related to dwarf traits in crape myrtle.**

| Name | Sequence (5’-3’) | Length | |
| --- | --- | --- | --- |
| M16337 | ACCAATGTCATAATTACTCCCGTGATAATAATGGTAGAACTCCATTGGAAGATTTGCAACAGTATCATAAGTTCCACCACGAGCATTCCCATCTGAGACTTGGTTCACAATTTCACCAGCATTGTCAGTTCCCGTGGCTCTAACCTCTTCAGTATCAGTAGTCTTGTCCTTTATTTCCATATTTTCAGGTTTATCATTGTTTGGATCTCTCGCCTCGGGTTTGGAGCTCTGAGTGGGTTTCCTAGACTCTTCGGCACTCATAATGGCATCCTTAGCAGCATCTGTATGAGCTGCAGCAATCGCCAAGCGCTCTTCTTCTTCAACTGCAGCTCGAACTCTCTCAAGAAACTTATCATCCTCTTCAGGGATAAAATGCCCTGCAAGAACAAATCTGGTAAGGAAACAACTTTCTAATGGAAAAAAATGGTTCAAGATGGGCAATCCACAATGACTCACATTGT | | 461 bp |
| M25207 | ACGTAGGTAGACAGACGTATACAGCATGGCCATAAAATGATCTTGGATCCATATGACACTCTTTTTTGCCAAGGGGATTGATCTCCTTATTACGAGGTTCTGTGCTTTATTCTTGATCTAGAATCTGTGATGCCGCTTCTCTTCTCACCGTTCTAGGCTCGTAAACCCTAAAATTTCTCTCCTTCGCTCACCATGAATTTGAATCTCTGTCAGCTGTCACCTTCAGCAAGTGTGGAAGCCAAAACAAATTGTTCTTTGTCAGAGGTGATACACGAGATTAGAACAAGACTCGGAAAAACCTATATATATATATATATAACTAATGAATAATATTTTCTAGATCCGTCCTATCAAATCCTATGTTTATTGGACATGAATATGTGGGGGATCGAGTTGTAGACTGCATGATTATTGTACGTACGATGTGCGTATATACCACGTGGCAATGTCAACGTAGT | | 458 bp |
| M38412 | ACAAATTAGAGGGATGCCACCTGAAGTTATTTCCCTCCAAAACATCCAACGAGCAGCATCCAAAGCTGGGGAGGTAATAGAAGTGGAATGGAAGGATTCAATTATGCCTAAATGGACATCGGTCCCCAAGGCCTTAGTCAAAGTCCAGGTTGGAGCACCGCTAGTTACTGGGTTTCAGATGAAAAGTAGCACGGGCAAGAAGCTGAAGATATATTTTAAATATGAAAAACTCTCAACTTTCTGTTATGATTGTGGCATCCTTGGGCATGAGCAAGGCATTTGTAATGCTGAAGGTCnGGTGGATCCCCATAAATATGGACCATGGCTGAGGTACGAGGAGAATGCCGATATACTCCCTCCGAATCTGGAACAACCTGAGAAGTTCGAGTCGCCGGAAAATCTCACCGGGAAAAGTTCCGGT | | 421 bp |
